# Left ventricular structural and functional changes in Friedreich ataxia – Relationship with body size, sex, age and genetic severity

**DOI:** 10.1371/journal.pone.0225147

**Published:** 2019-11-13

**Authors:** Roger E. Peverill, Giovanni Romanelli, Lesley Donelan, Rhonda Hassam, Louise A. Corben, Martin B. Delatycki

**Affiliations:** 1 Monash Cardiovascular Research Centre and Department of Medicine (School of Clinical Sciences at Monash Medical Centre), Monash University and Monash Health, Clayton, Victoria, Australia; 2 Bruce Lefroy Centre for Genetic Health Research, Murdoch Childrens Research Institute, Royal Children’s Hospital, Parkville, Victoria, Australia; 3 Victorian Clinical Genetics Services, Royal Children’s Hospital, Parkville, Victoria, Australia; Heart and Diabetes Center NRW, UNiversity Hospital of the Ruhr-University Bochum, GERMANY

## Abstract

**Introduction:**

Although a concentric pattern of left ventricular (LV) geometry appears to be common in Friedreich ataxia (FRDA), there is no accepted method for diagnosing LV abnormalities in FRDA, sex and body size have often not been taken into consideration, and it has not been clear whether children and adults should be classified using the same criteria. The aim of this study was to better define the LV geometric changes in FRDA with respect to sex, body size and subject age, and to investigate the relationship of LV changes with genetic severity, as assessed by GAA repeat length within the shorter allele of the *FXN* gene (GAA1).

**Methods:**

Echocardiography was performed in 216 subjects (68 children, 148 adults), measurements were made at end-diastole of LV internal diameter (LVEDID), septal wall thickness (SWT), LV length (LVEDL) and LV volume (LVEDV), and calculations were made of relative wall thickness (RWT), LV mass and LV ejection fraction (LVEF).

**Results:**

The most common LV abnormalities in both adults and children with FRDA were increases in RWT and age-normalized RWT. In adults with a normal LVEF, all LV variables other than RWT were larger in males independent of body surface area (BSA), and all LV variables other than SWT and RWT were positively correlated with BSA. After adjustment for sex and BSA, GAA1 was a positive correlate of SWT and RWT (but not of LV mass), and was an inverse correlate of LVEDID, LVEDL and LVEDV. In children with a normal LVEF, SWT, LV mass and LVEDL were larger in males than females after adjusting for BSA, and in combination with sex, BSA was a positive correlate of all the LV variables except SWT and RWT. In children there were no correlations of GAA1 with any of the LV variables.

**Conclusion:**

In FRDA, increases in RWT and age-normalized RWT are the most frequent LV structural abnormalities, sex and body size are important determinants of most other LV structural variables in both children and adults, and increased genetic severity is associated with a smaller left ventricle and increased LV wall thickness in adults, but not associated with LV size or wall thickness in children.

## Introduction

Friedreich ataxia (FRDA) is an autosomal recessive neurodegenerative disease caused by a defect in the gene *FXN* which encodes for the mitochondrial protein frataxin [1]. In approximately 96% of individuals there is homozygosity for a GAA expansion in intron 1 of *FXN*, and in the remainder there is a deletion or point mutation within one of the alleles and a GAA expansion in the other allele. Cardiac disease is a frequent accompaniment of FRDA and not only can lead to arrhythmias and cardiac failure, but has been reported to be the most common cause of death [2, 3]. LV structural changes in FRDA have been documented in a number of studies, with increase in LV wall thickness and reduction in LV chamber size being commonly reported abnormalities [4–11]. It is also well recognized that some subjects with FRDA develop a reduced LV ejection fraction and increased LV size, and there is some evidence to suggest that this follows a progression from an earlier stage in which there was increased wall thickness [12]. Nevertheless, questions remain about the nature and frequency of the patterns of cardiac involvement in FRDA.

The number of GAA repeats in the shorter allele of the *FXN* gene (GAA1) is inversely related to cellular levels of frataxin [13, 14], and there has therefore been interest in the ability of GAA1 to explain disease severity in FRDA. There have been reports that GAA1 is associated with earlier age at the onset of neurological symptoms (AOS) [15], increases in LV wall thickness [4, 5, 9, 10, 16, 17], relative wall thickness (RWT) [18], LV mass [5, 19] and LV mass index [4, 20] and a smaller LV end-diastolic internal diameter (LVEDID) [17]. However, the presence and extent of the associations of GAA1 with cardiac variables have varied considerably between the studies, and two of the largest studies did not find a relationship of GAA1 with the extent of cardiac involvement [8, 9]. In addition, there have also been reports that the length of GAA repeats on the larger allele (GAA2) is associated with AOS [17, 21–24], the presence of cardiac involvement [21, 24], the extent of septal wall thickness (SWT) [4, 6, 17] and LV mass index [4]. However, GAA2 appears to be less closely associated than GAA1 with AOS [17, 21, 23] and cardiac variables [4, 17, 21], and whether GAA2 provides information about disease severity in FRDA independently of GAA1 has not been specifically investigated.

There are factors independent of the disease process in subjects with FRDA which are likely to influence LV cavity size, wall thickness and LV mass, and may need to be taken into account when investigating the LV structural and functional changes in FRDA. First, all LV structural variables are normally dependent on body size and while there may not be an ideal method for indexation to body size [25], not indexing is likely to be an inferior option. Clarification of this issue in FRDA is important because a number of previous studies which have investigated the relationships of GAA in FRDA have used non-indexed LV variables [5, 6, 8, 12, 17]. Second, in healthy individuals there are different relationships between LV structural variables and body size in adults [19] compared to children [26], however, in many cases children and adults have been combined together in analyses of LV structure and function in FRDA [4, 8, 9, 10, 12, 17]. Third, sex needs to be considered, as male sex is associated with greater LV mass independent of body size in healthy adults and older children [27], and males and females have been shown to differ in the nature and extent of LV hypertrophy and function which develop in various pathological states [28, 29]. Clarification of the role of sex effects on LV structure in FRDA is particularly important as sex has often not been taken into consideration when making a diagnosis of LV hypertrophy in FRDA [4, 8, 17].

Accordingly, the main aims of this cross-sectional study in FRDA subjects were (1) to investigate the relationship of LV variables with various measures of body size and to compare these methods in children and adults, (2) to determine the effects of sex after adjustment for body size on LV variables in children and adults, and (3) to determine the relationships of GAA1 and GAA2 with LV structural variables in children and adults after appropriate adjustment for other associated variables.

## Methods

### Study group

The study design was approved by the Monash Health Human Research and Ethics committee (RES-18-0000-732Q) and all clinical investigation was conducted according to the principles expressed in the Declaration of Helsinki. The need to obtain consent from adult subjects, or consent from the parent or guardian of subjects who were minors, was waived for this retrospective study of clinically indicated testing. We identified 216 subjects with genetically confirmed FRDA who had attended the multidisciplinary FRDA clinic at Monash Medical Centre, Monash Health and had undergone echocardiography.

### Echocardiography

Transthoracic echocardiography was performed using a Sonos 5500 ultrasound machine (Philips, Amsterdam, The Netherlands) in 131 subjects, a Vivid 7 (GE Healthcare, Chicago, IL, USA) in 79 subjects, an E9 (GE Healthcare, Chicago, IL, USA) in 2 subjects and an IE33 (Philips, Amsterdam, The Netherlands) in 4 subjects. The vast majority of studies (>90%) were performed by one of two experienced sonographers (LD & RH) using a standardized protocol. M-mode images of the left ventricle were obtained in the parasternal long axis and short-axis views just distal to the mitral valve leaflet tips after alignment of the cursor perpendicular to the LV wall. Two dimensional images were used to facilitate identification of the endocardium. Measurements were performed off-line using Xcelera V1.2 L4 SP2 (Philips), including standard M-mode measurements of LV SWT, posterior wall thickness, LVEDID and LV end-systolic diameter [30]. In subjects in whom on-axis M-mode images could not be obtained, caliper measurements in the parasternal views were performed, with cross checking of the results when possible with caliper measurements made using the apical and subcostal four-chamber views.

LV end-diastolic external diameter was calculated by adding LVEDID to SWT and posterior wall thickness, as described by Stoylen et al [31]. Fractional shortening (FS) was calculated as (LVEDID-LV end-systolic diameter)/LVEDID. RWT was calculated as 2 times the posterior wall thickness divided by the LVEDID, and elevation of RWT was diagnosed if the RWT was >0.42 [30]. Given that RWT increases with age in both children and adults [26, 31], an age-normalized RWT was also calculated by a previously described method, and elevation of age-normalized RWT was diagnosed in children if >0.39, and in adults if >0.42 [26]. LV mass was calculated using the modified formula of Devereux et al [32] and indexation was performed to both body surface area (BSA) in m^2^ and height^2.7^ [33]. Sex-specific cutoffs for elevation of LV mass index with indexation to BSA were used for males (>115 g/m^2^) and females (>95 g/m^2^) [30], and age-specific cutoffs for elevation of LV mass index with indexation to height^2.7^ were used for male adults (>50 g/height^2.7^), female adults (>47 g/height^2.7^), male children (>44 g/height^2.7^), and female children (>40 g/height^2.7^) [33]. In children, z-scores were calculated for LVEDID, SWT, posterior wall thickness and LV mass, as described by Kampmann et al [34].

Four- and 2-chamber loops of LV contraction were recorded and used for measurement of LV end-diastolic volume (LVEDV) and LV end-systolic volume, and the calculation of LVEF using the biplane method of discs. LV end-diastolic length (LVEDL) was generated during the automatic measurements provided during volume and LVEF calculations in the 2- and 4-chamber views, and the longest length from the 2-chamber and 4-chamber views has been used in the analysis [35].

LV inflow velocities were recorded using pulsed-wave Doppler in the apical 4-chamber view with the sample volume located at the level of the mitral leaflet tips. The peak velocity (E) and deceleration time of early diastolic filling and the peak velocity (A) of the atrial phase of LV diastolic filling were measured.

### Genetic analysis

The presence and size of the GAA expansion on both alleles of *FXN* was established by PCR analysis, as previously described [36].

### Statistics

Statistical analysis was performed using Systat V13 (Systat Software, Chicago, IL, USA). Results are expressed as mean ± SD, or median (range) if not normally distributed. The relationships of standard LV measurements with anthropometric data, sex, age and the disease specific variables of GAA1, GAA2, AOS and symptom duration was analyzed using linear regression analysis in subjects who had a normal LVEF, were homozygous for GAA repeat expansions and had GAA measurements made at our institution. Univariate and multivariate analyses of LV measurements were performed separately in adults and children. The generalized linear model was used for multivariate analyses which combined continuous with categoric variables such as sex, or child versus adult. BSA was entered first in all models and a contribution from sex was considered as the second step. GAA1 or GAA2 were added as a third step, starting with whichever variable had the higher r value on univariate analysis. Age was added as a fourth step in the model and AOS was added next if it was a significant correlate of the dependent variable on univariate analysis.

## Results

Of the 216 subjects with FRDA who had an echocardiogram performed during the study period, 9 had *FXN* point mutations, 202 were homozygous for GAA repeats and had results available from our laboratory, and 5 were known to be homozygous for GAA repeats on the basis of testing at other institutions. There were 9 subjects with a diagnosis of diabetes. The clinical and available genetic data regarding GAA1 and GAA2 of the total group divided into children (≤18 years) and adults (>18 years) are shown in Table 1. GAA1 and GAA2 were available in 64 children and 138 adults. In children there was a positive correlation of GAA1 with GAA2 (r = 0.69, p<0.001), an inverse correlation of GAA1 with AOS (r = -0.34, p = 0.008), but no correlation of GAA2 with AOS (r = -0.18, p = 0.18). In contrast, in adults there was a weaker positive correlation of GAA1 with GAA2 (r = 0.34, p<0.001), a stronger inverse correlation of GAA1 with AOS (r = -0.62, p<0.001), and there was an inverse correlation of GAA2 with AOS (r = -0.24, p = 0.007). However, only GAA1 remained a significant predictor of AOS (p<0.001) in adults when GAA1 and GAA2 were both combined in a multivariate model.

**Table 1:** Demographic, anthropometric and disease specific characteristics in adults and children with Friedreich ataxia.

	Children	Adults
n	68	148
Male / Female	44/24	72/76
Age (years)	14.3±3.3	34.7±11.6
Height (cm)	158±16	170±10
Weight (kg)	47.5±15.2	69.6±14.4
BSA (m^2^)	1.47±0.30	1.80±0.21
Body mass index (kg/m^2^)	18.7±3.9	23.9±4.3
Heart rate (/min)	75±12	74±12
Systolic blood pressure (mmHg)	112±17	116±16
Diastolic blood pressure (mmHg)	62±9	72±10
GAA1*	734±198	609±218
GAA2*	893±175	870±216
AOS (years)	9.1±4.3	16.5±8.3
Symptom duration (years)	4.9±3.8	17.4±10.9

BSA–body surface area, AOS—age at onset of neurological symptoms, GAA1—number of GAA repeats in the shorter allele of the *FXN* gene, GAA2—number of GAA repeats in the longer allele of the *FXN* gene; * GAA1 and GAA2 were available in 64 children and 138 adults.

There were 2 adults in atrial fibrillation, 2 adults with a pacemaker and the rest of the cohort were in sinus rhythm. There were 15 individuals with a LVEF ≤50%, of whom three were children. Characteristics of the 12 adults with a LVEF ≤50% included an age of 31±10 years, one subject with a point mutation, GAA1 of 578±223, GAA2 of 968±148 in the other 12 subjects, AOS of 10 (5–29) years and symptom duration of 13 (4–30) years.

**Fig 1:**
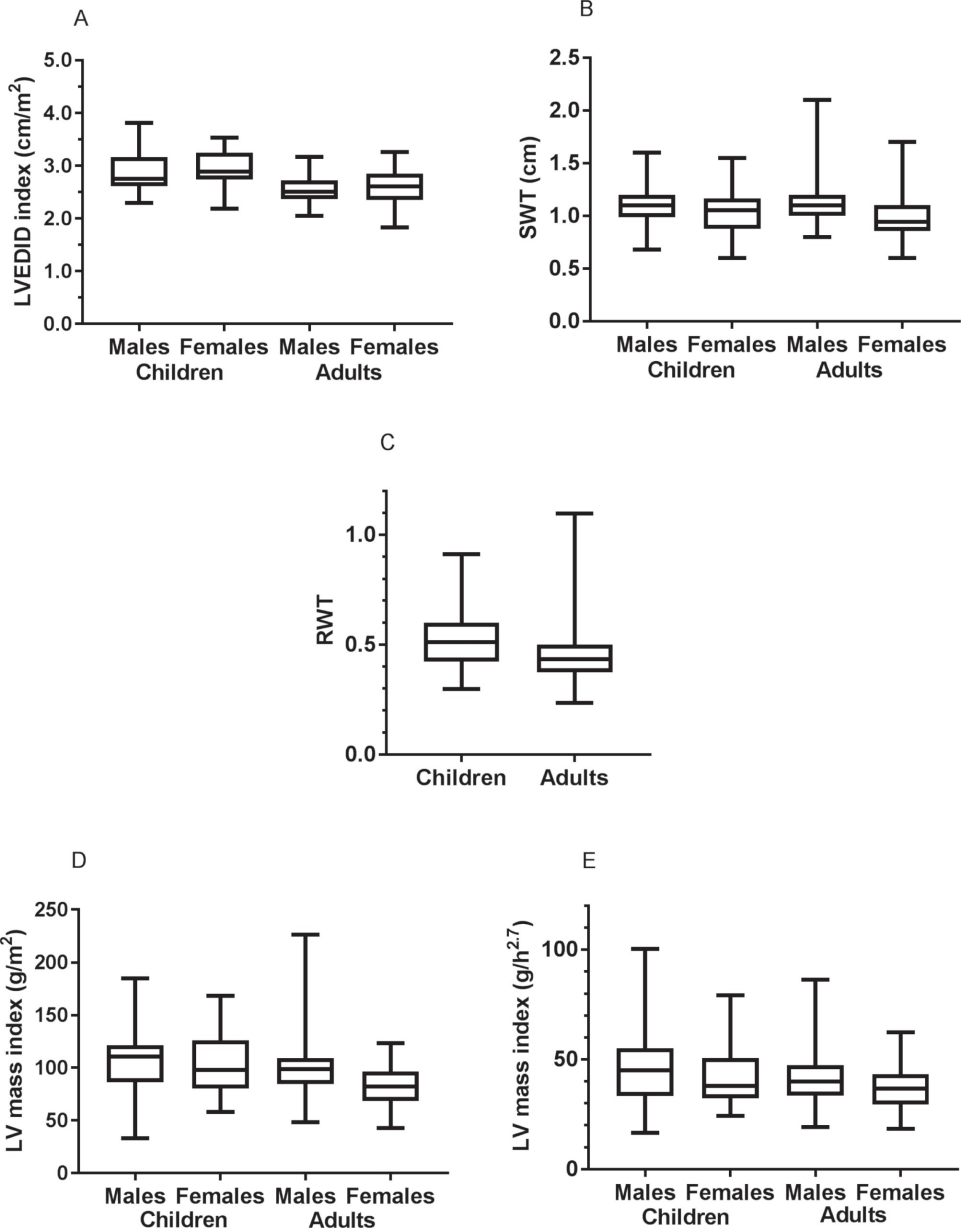
Box and whisker plots of left ventricular structural variables. Box and whisker plots for subjects with Friedreich ataxia who were homozygous for GAA repeat expansions, had GAA measurements performed in our laboratory, and had sinus rhythm and a LVEF >50%, showing LVEDID indexed to BSA (A), SWT (B), RWT (C), LV mass indexed to BSA (D) and LV mass indexed to height^2.7^ (E). Children and adults are shown separately for all variables, and males and females are shown separately for variables in which male sex was associated with larger variable size independent of BSA. See Table 2 for abbreviations.

All subsequent analyses have been performed on the group of subjects who were homozygous for GAA repeat expansions, had GAA measurements performed in our laboratory, and had sinus rhythm and a LVEF >50%. The LV and transmitral Doppler variables of subjects who fulfil these conditions are shown in Table 2, with children and adults shown separately. Box and whisker plots for selected indexed LV structural variables in these subjects are shown in Fig 1, with children and adults shown separately, and males and females shown separately as appropriate.

**Table 2:** Left ventricular and transmitral Doppler variables in children and adults with FRDA who were homozygous for GAA repeat expansions and had a normal LVEF.

	Children	Adults
n	60	124
LDEDID (cm)	4.2±0.5	4.6±0.5
SWT (cm)	1.1±0.2	1.0±0.2
Posterior wall thickness (cm)	1.1±0.2	1.0±0.2
Fraction shortening (%)	40.1±8.3	39.5±7.5
RWT	0.52±0.13	0.46±0.12
Age-normalized RWT	0.52±0.13	0.47±0.12
LV end-diastolic external diameter (cm)	6.3±0.6	6.7±0.6
LV mass (g)	152±46	168±52
LV mass index (g/m^2^)	107±31	93±27
LV mass index (g/m^2^)–Male	111±31	105±29
LV mass index (g/m^2^)–Female	100±31	84±20
LV mass index (g/height^2.7^)	45±15	40±11
LV mass index (g/height^2.7^)—Male	46±15	43±12
LV mass index (g/height^2.7^)—Female	43±16	37±10
LVEDL (cm)	8.2±0.9	8.3±0.8
LVEDV (mL)	84±27	86±30
LVEF (%)	65±6	64±7
E (cm/s)	82±12	76±14
A (cm/s)	44±11	46±14
E/A	1.98±0.57	1.85±0.84
Deceleration time (ms)	157±30	169±38

LV–left ventricular, LVEDID—left ventricular end-diastolic internal diameter, SWT—septal wall thickness, RWT—relative wall thickness, LVEDL—left ventricular end-diastolic length, LVEDV—left ventricular end-diastolic volume, LVEF—left ventricular ejection fraction, E—peak early diastolic transmitral flow velocity, A—peak late diastolic transmitral flow velocity

### LV variables in children homozygous for GAA repeat expansions and a normal LVEF

In homozygote children who were in sinus rhythm with a LVEF >50% (n = 60), the RWT was increased (>0.42) in 80%, the age-normalized RWT was increased (>0.39) in 83%, whereas the sex specific LV mass index (g/m^2^) was increased in only 47% and the sex specific LV mass index (g/height^2.7^) was increased in only 48%. There were 83% children who had at least one abnormality based on the above LV structural criteria. The mean Z-scores for LVEDID and LV end-systolic diameter were negative (-0.26±1.15 cm and -0.56±1.59 cm, respectively) and the mean Z-scores for SWT, posterior wall thickness and LV mass were positive (4.20±2.56, 3.07±1.79 and 1.34±1.47, respectively).

On univariate analysis, there were positive correlations of LVEDID, LV end-diastolic external diameter, LV mass, LVEDL and LVEDV with all the anthropometric variables, but no correlations of SWT with any of the anthropometric variables. RWT and age-normalized RWT were inversely correlated with all the anthropometric variables (Table 3). Male sex was associated with a higher LV end-diastolic external diameter, LV mass and LVEDL (p<0.01 for all), a borderline increase in SWT (p = 0.063) and posterior wall thickness (p = 0.051), but there was no difference between males and females for LVEDID, RWT, age-normalized RWT, LV mass index (g/m^2^ or g/h^2.7^) or LVEDV (p>0.01 for all). RWT and age-normalized RWT were both inversely correlated with age and AOS, and age-normalized RWT, but not RWT, was positively correlated with symptom duration. None of the LV variables were correlated with either GAA1 or GAA2. In contrast to the normal pattern of a positive correlation between LVEDID and SWT seen in healthy children [26], LVEDID was inversely correlated with SWT (r = -0.31, p = 0.012).

**Table 3:** Univariate correlations (r) of left ventricular structural variables in children with FRDA who were homozygous for GAA repeat expansions and had a normal LVEF (n = 60).

	LVEDID	SWT	RWT	Age-normalized RWT	LV end-diastolic external diameter	LV mass	LVEDL	LVEDV
Height	0.70	-0.07	-0.32	-0.33	0.62	0.43	0.65	0.65
Weight	0.75	0.01	-0.32	-0.30	0.69	0.54	0.65	0.65
BSA	0.75	-0.06	-0.36	-0.35	0.65	0.47	0.64	0.67
GAA1	-0.23	0.02	0.16	0.12	-0.18	-0.09	-0.12	-0.20
GAA2	-0.20	-0.07	0.08	0.03	-0.21	-0.19	-0.12	-0.10
Age	0.60	-0.13	-0.30	-0.27	0.48	0.32	0.63	0.62
AOS	0.55	-0.17	-0.34	-0.30	0.39	0.23	0.43	0.46
Symptom duration	-0.16	0.24	0.20	0.22	0.06	0.09	-0.02	-0.04

See Tables 1 and 2 for abbreviations

Multivariate analyses were performed in homozygote children in sinus rhythm with a normal LVEF. For those dependent variables which were positively correlated with body size, modelling began with the addition of sex to BSA and then with the sequential addition of GAA1, GAA2 and age. Only variables that remained statistically significant during this process are shown in the models in Table 4. LV end-diastolic external diameter, LV mass and LVEDL (p<0.02 for all), but not LVEDID or LVEDV, were larger in males than females after adjusting for BSA. In combination with sex, BSA was a positive correlate of LVEDID, LV end-diastolic external diameter, LV mass, LVEDL and LVEDV (p<0.001 for all). In multivariate models with adjustment for sex and BSA, there were no contributions to LVEDID, SWT, posterior wall thickness, LV end-diastolic external diameter, LV mass, LVEDL or LVEDV from GAA1, GAA2, age or AOS. There were no independent correlates of RWT or age-normalized RWT in multivariate models which included various combinations of BSA, age and AOS.

**Table 4:** Multivariate models of left ventricular structural variables in children with FRDA who were homozygous for GAA repeat expansions and had a normal LVEF (n = 60).

		LVEDID	LVEDED	LV mass	LVEDL	LVEDV
BSA	β	0.74	0.63	0.44	0.60	0.67
	p	<0.001	<0.001	<0.001	<0.001	<0.001
Sex	p		0.019	0.006	0.011	
Adjusted r^2^		0.53	0.47	0.31	0.45	0.44

See Tables 1 and 2 for abbreviations

### LV variables in adults homozygous for GAA repeat expansions and a normal LVEF

In homozygous adult subjects in sinus rhythm with a LVEF>50% (n = 124), the RWT was increased (>0.42) in 57%, the age-normalized RWT was increased (>0.42) in 61%, whereas the sex specific LV mass index (g/m^2^) was increased in only 24%, and the sex specific LV mass index (g/height^2.7^) was increased in only 18%. At least one abnormality based on the above criteria was present in 65%. On univariate analysis (Table 5), LVEDID, LV end-diastolic external diameter, LV mass, LVEDL and LVEDV were positively correlated with all of the anthropometric measures, there were positive correlations of SWT and posterior wall thickness with height, but not with weight or BSA, and there were no correlations of RWT or age-normalized RWT with anthropometric measures. All of the LV variables, other than RWT and age-normalized RWT, were larger in males than females (p<0.01 for all), even after adjustment for height or BSA (p<0.01 for all). After adjustment for sex, BSA remained a significant correlate of LVEDID, LVEDL and LVEDV (p<0.01 for all), and also remained a borderline significant correlate of LV mass (p<0.05), but was no longer a correlate of LV end-diastolic external diameter. After adjustment for sex, height was no longer a correlate of SWT or posterior wall thickness. In contrast to the normal positive correlation between LVEDID and SWT seen in healthy adults [26], there was no significant correlation of LVEDID with SWT, and the r value was negative (r = -0.14, p = 0.10). GAA1 was a positive correlate of RWT and age-normalized RWT, an inverse correlate of LVEDID, LVEDL and LVEDV, but was not a correlate of SWT, posterior wall thickness, LV end-diastolic external diameter or LV mass. GAA2 was not a significant correlate of any of the LV variables.

**Table 5:** Univariate correlations of left ventricular structural variables in adults with FRDA who were homozygous for GAA repeat expansions and had a normal LVEF (n = 124).

	LVEDID	SWT	Posterior wall thickness	RWT	Age-normalized RWT	LV end-diastolic external diameter	LV mass	LVEDL	LVEDV
Height	0.39	0.20	0.26	0.02	0.02	0.47	0.43	0.52	0.41
Weight	0.49	0.06	0.08	-0.17	-0.19	0.42	0.35	0.45	0.46
BSA	0.52	0.13	0.12	-0.12	-0.13	0.50	0.43	0.53	0.50
GAA1	-0.31	0.17	0.11	0.23	0.26	-0.14	-0.06	-0.29	-0.33
GAA2	-0.11	0.15	0.13	0.14	0.14	0.01	0.03	-0.12	-0.07
Age	-0.07	-0.16	-0.18	-0.14	-0.28	-0.19	-0.18	-0.07	-0.11
AOS	0.15	-0.16	-0.14	-0.20	-0.26	0.02	-0.03	0.15	0.16
Symptom duration	-0.14	-0.08	-0.09	-0.04	-0.11	-0.19	-0.16	-0.18	-0.19

See Tables 1 and 2 for abbreviations

Multivariate models of LVEDID, LV end-diastolic external diameter, LV mass, LVEDL and LVEDV were constructed with the sequential addition of BSA, sex, GAA1, GAA2 and age in homozygote adult subjects with sinus rhythm, a normal LVEF and GAA results available from our laboratory. Only independent variables which remained significant during this stepwise process are shown in the final models (Table 6). In conjunction with BSA and sex, GAA1 was inversely correlated with LVEDID, LV end-diastolic external diameter, LVEDL and LVEDV (p<0.01 for all), but not with LV mass. In multivariate models which also added age, LVEDID, LV end-diastolic external diameter, LVEDL and LVEDV behaved similarly, being larger in males, positively correlated with BSA, and inversely correlated with both GAA1 and age. At least 1/3 of the variances in LVEDID, LV end-diastolic external diameter, LV mass, LVEDL and LVEDV could be accounted for by combinations of BSA, sex, GAA1 and age. SWT was positively correlated with sex and GAA1. None of SWT, posterior wall thickness or RWT were independently correlated with age. GAA1 was the only independent correlate of RWT, whereas age-normalized RWT was positively correlated with GAA1 and inversely correlated with age. In multivariate models, GAA2 was not correlated with any of the LV variables, and this was tested in the absence and presence of GAA1.

**Table 6:** Multivariate models of left ventricular structural variables in adults with FRDA who were homozygous for GAA repeat expansions and had a normal LVEF (n = 124).

		LVEDID	SWT	RWT	Age-normalized RWT	LV end- diastolic external diameter	LV mass	LVEDL	LVEDV
BSA	β	0.35				0.30	0.18	0.32	0.35
	p	<0.001				<0.001	0.042	<0.001	<0.001
Sex	p	0.009	<0.001			<0.001	<0.001	<0.001	0.023
GAA1	β	-0.27	0.20	0.23	0.19	-0.15		-0.25	-0.31
	p	0.001	0.023	0.012	0.033	0.035		0.001	<0.001
Age	β	-0.15			-0.23	-0.24	-0.19	-0.17	-0.21
	p	0.056			0.013	<0.001	0.01	0.02	0.008
Adjusted r^2^		0.36	0.12	0.04	0.10	0.45	0.36	0.41	0.36

See Tables 1 and 2 for abbreviations

### Transmitral Doppler variables in individuals homozygous for GAA repeat expansions and a normal LVEF

In children, there were no significant correlations of either E or A with age, sex, GAA1 or GAA2. In adults, on multivariate analysis, E was inversely correlated with age (β = -0.27, p = 0.002) and GAA1 (β = -0.25, p = 0.004), and was smaller in males than females (p = 0.002), whereas A was positively correlated with age (β = 0.35, p<0.001) and inversely correlated with GAA1 (β = -0.25, p = 0.003), but not affected by sex.

### Multivariate analyses in combined group of children and adults homozygous for GAA repeat expansions and a normal LVEF

Selected multivariate analyses were performed in the combined group of children and adults with a normal LVEF and homozygous for GAA repeat expansions (n = 184) to allow comparison with previous studies, and also to investigate whether there could be statistical differences between children and adults in the relationship of LV variables with GAA1 and GAA2. GAA1 was correlated with GAA2 in this combined group (r = 0.43, p<0.001) and both GAA1 and GAA2 were inverse correlates of AOS (r = -0.60 & r = -0.19, respectively, p<0.01 for both), but when GAA1 and GAA2 were both entered in a model of AOS only GAA1 remained a significant correlate (p<0.001). In models which included sex, BSA and age, the addition of either GAA1 or GAA2 contributed to the prediction of LVEDID and LVEDL, but only GAA1 contributed to the prediction of LVEDV (Table 7). However, GAA2 made no independent contribution to the models of LVEDID or LVEDL in which GAA1 was also included. SWT and posterior wall thickness were both larger in males (p<0.01 for both) but were not independently related to BSA, age, GAA1 or GAA2. After adjustment for sex, BSA, age and GAA1, there was no statistical difference between children and adults for LVEDID or LVEDL, but there was a trend for a lower LVEDV in adults (p = 0.06). Independent predictors of a higher E were female sex (p = 0.005), younger age (p<0.001) and smaller GAA1 (p = 0.011), and there was no statistical difference between children and adults. Independent predictors of a higher A were older age (p<0.001), lower GAA1 (p = 0.001) and higher heart rate (p<0.001), but in contrast to E, being a child was a predictor of a higher A (p = 0.03) independent of age, heart rate and GAA1. GAA2 was not a significant contributor to models of E or A in the combined group in the absence or presence of GAA1.

**Table 7:** Multivariate models of LVEDID, LVEDL and LVEDV in combined group of children and adults with FRDA who were homozygous for GAA repeat expansions and had a normal LVEF (n = 184).

Dependent variable	Independent variable	β	p	Adjusted r^2^
LVEDID	Sex		0.028	
	BSA	0.62	<0.001	
	Age	-0.13	0.058	
	GAA1	-0.22	<0.001	0.49
LVEDID	Sex		0.024	
	BSA	0.66	<0.001	
	Age	-0.08	0.30	
	GAA2	-0.14	0.007	0.44
LVEDL	Sex		<0.001	
	BSA	0.50	<0.001	
	Age	-0.23	0.001	
	GAA1	-0.18	0.004	0.44
LVEDL	Sex		<0.001	
	BSA	0.55	<0.001	
	Age	-0.18	0.006	
	GAA2	-0.15	0.006	0.44
LVEDV	Sex		<0.001	
	BSA	0.50	<0.001	
	Age	-0.28	<0.001	
	GAA1	-0.26	<0.001	0.37
LVEDV	Sex		<0.001	
	BSA	0.54	<0.001	
	Age	-0.20	0.006	
	GAA2	-0.10	0.09	0.33

See Tables 1 and 2 for abbreviations

## Discussion

There are a number of new findings from the present study regarding LV structural features in FRDA. (1) The most common abnormal LV findings in FRDA in both children and adults in our cohort were increases in RWT and age-normalized RWT. (2) In separate analyses of children and adults with FRDA, body size was an independent determinant of all the measures of LV size and LV mass other than wall thickness, RWT and age-normalized RWT in both age groups. (3) Male sex was a determinant of a larger magnitude of all the measures of LV structure other than RWT and age-normalized RWT in adults with FRDA, and this effect was independent of body size. Male sex was also an independent determinant of larger SWT, LV end-diastolic external diameter, LV mass and LVEDL in children with FRDA, but was not an independent determinant of LVEDID, RWT or age-normalized RWT. (4) In adults with FRDA, GAA1 was an independent determinant of both smaller LV size and increased wall thickness, and possibly because of this combination, was not a determinant of LV mass. In contrast, there were no associations of GAA1 with LV size or wall thickness seen in children. (5) GAA2 was positively correlated with GAA1 in both children and adults, but was not an independent predictor of any of the LV variables in either children or adults. (6) Increasing age in adults with FRDA and a preserved LVEF was independently associated with decreases in LV size and LV mass, however, after adjustment for body size and sex there was no effect of age on measures of LV size or LV mass in children.

That LV size and mass are determined in part by body size is well recognized from studies in healthy adults [37, 38], and the relation between LV size and body size may be of even greater importance in children due to the positive associations between the growth in the body and the size of LV structures during childhood [34, 39, 40]. Consistent with this, in the present study in subjects with FRDA an association between most LV variables and body size was demonstrated in both children and adults. The exceptions were LV wall thickness, which was not related to body size, presumably related to any effects of body size being outweighed by the pathological effects of the disease process which lead to an increase in LV wall thickness, and RWT, which was not expected to be related to body size because it involves an adjustment for cardiac size within the individual. The present study has not been able to address the most appropriate means of LV indexation in FRDA as this is not only likely to vary for the different LV structural measurements for reasons of dimensionality, but could turn out to be an allometric relationship with lean body mass [25], which was not measured in the present study. Nevertheless, the findings of the present study suggest that inclusion of a measurement of body size is essential in investigations of the determinants of LV structural change in FRDA and also suggest the need for reevaluation of findings from previous studies in FRDA in which body size may not have had appropriate consideration in the analyses [4, 5, 17].

Previous studies in healthy adults have generally shown that male sex is associated with larger magnitudes of all LV variables (other than RWT), and that at least part of this sex effect is independent of body size [37, 38]. Consistent with this, in adults with FRDA in the present study, sex was an independent determinant of all LV variables apart from RWT. Studies investigating the effect of sex on LV variables in healthy children have often found no or minimal effects, but it is possible that these studies, which generally included ages from infancy to 18 years [34, 39, 40], may have missed seeing effects of sex which are likely to be confined to post pubertal children. Importantly, in children with FRDA of age range from 4–18 years in the present study, male sex was an independent determinant of larger SWT, LV end-diastolic external diameter, LV mass and LVEDL. Similar to body size, sex has not always been a consideration in FRDA studies which have investigated the effects of FRDA on LV size and mass. In particular, a number of studies have made a diagnosis of LV hypertrophy in FRDA using Henry’s nomograms [4, 8, 17, 41], first described in 1980 at a time before the effects of male sex on the left ventricle were recognized, and which do not take sex into consideration. A diagnosis of the absence of cardiomyopathy based on a normal LV wall thickness using Henry’s nomograms not only has the intrinsic limitation of lacking consideration of sex effects, but it has also been demonstrated in previous FRDA studies to fail in identifying an increase in RWT in a proportion of subjects. Thus, Weidemann et al reported a RWT of 0.42±0.08 in a subgroup of 64/205 children and adults who were classified to be free of cardiomyopathy, this result indicating that a substantial proportion of the subjects in this subgroup would have had an abnormal RWT of 0.43 or above [8]. Similarly, in a subgroup of 56/133 adults and children who were classified as not having LV hypertrophy, Pousset et al reported a RWT of 0.43±0.07 [17]. These anomalies can be attributed to misclassification by Henry's nomograms, which in turn can, at least in part, be attributed to lack of consideration of sex effects on LV structure.

There are limitations of using a RWT of >0.42 to define LV remodeling in a mixed group of children and adults, not only because the normal range of RWT differs between adults and children, but also because RWT increases with age in both children and adults [26, 31]. In an attempt to address this, age-normalized RWT was categorized in adults and children separately in the present study using a previously described method [26]. However, the use of age-normalized RWT only identified a slightly greater percentage of abnormal hearts than RWT. Thus, in subjects with a normal LVEF, the prevalence in children of an increase in RWT was 80% and of an increase in age-normalized RWT was 83%, and in adults the prevalence of an increase in RWT was 57% and of an increase in age-normalized RWT was 61%. Both RWT and age-normalized RWT were considerably more sensitive than LV mass index for detecting a LV abnormality in FRDA in both children and adults, irrespective of whether indexing was performed to BSA or h^2.7^, and despite the use of sex-specific cutoffs.

The relationships of GAA1 with LV variables have been evaluated in a number of previous studies but there has been considerable divergence in the reported findings [4–6, 8, 9, 17, 21, 24, 42]. In the present study, in the adult group after adjustment for sex and BSA, there were inverse correlations of GAA1 with all the measures of LV cavity size, these being LVEDID, LVEDL and LVEDV. There were positive correlations of GAA1 with SWT in adults after adjusting for sex and there was also a positive correlation of GAA1 with RWT. In contrast, there was no correlation of GAA1 with either LV mass or LV mass index and no correlations of GAA1 with any of the LV variables in children. One possible explanation for the apparent discrepancies with previous studies is that a number of studies have combined adults and children in the analysis. This may be important for reasons already discussed above regarding body size and sex, but also may reflect intrinsic differences between the associations of the cardiac phenotype in children and adults. Also consistent with the proposition that the relationships in children may be different are the finding that there were no correlations of GAA1 with LV structure in a FRDA study where the majority of the subjects were children [9]. In the present study (where adults comprised more than 2/3 of the cohort), when adults and children were analyzed together the correlations with GAA1 were similar to those seen in the adult group. Importantly, that there may be intrinsic differences between children and adults could not be confirmed by this study, but was suggested by the findings that there was a trend for a lower LVEDV and a lower A in adults compared to children, even after adjustment for age and GAA1.

LV end-diastolic external diameter was calculated in FRDA for the first time in this study and in contrast to healthy aging where older age is accompanied by increases in both RWT and LVEDED [31], higher GAA1 was accompanied by increased RWT but reduced LVEDED. This finding, in combination with the inverse association of GAA1 with LV cavity size, demonstrates that a negative LV remodeling process occurs in FRDA in parallel with the increase in wall thickness. A similar process has also been described for the autosomal dominant hypertrophic cardiomyopathies [43]. Negative LV remodeling in association with increased wall thickness is also a large part of the explanation why increased LV mass index is less common than increased RWT in both adults and children with FRDA, and also why assessment using Henry’s nomograms, which are based on wall thickness alone, can fail to identify an abnormality in subjects with FRDA and an increase in RWT.

GAA2 was positively correlated with GAA1 in both adults and children in the present study, and was also an inverse correlate of AOS in adults, but it was not a correlate of AOS in children, was not a correlate of AOS in adults independent of GAA1, and was not a correlate of any of the LV structural variables when children and adults were analyzed separately. There have been previous reports that GAA2 is associated with AOS [17, 21–24] and cardiac involvement in FRDA [4, 4, 6, 17, 21, 24] and indeed, when children and adults were analyzed together in the present study, GAA2 was an inverse correlate of LVEDID and LVEDL, independent of sex, BSA and age. However, this may be the first study to report that GAA1 and GAA2 are also correlated, with this providing a potential explanation for GAA2 associations in previous reports which does not entail a direct relationship between GAA2 and the phenotype. Indeed, GAA2 was no longer a significant predictor of either LVEDID or LVEDL in the combined group when GAA1 was also included in multivariate models. There is no current explanation for the correlation of GAA1 with GAA2. While there is one study showing associations of both GAA1 and GAA2 with frataxin levels [44], this study did not investigate whether these associations were independent.

Understanding the nature of LV remodeling in FRDA is made more difficult given there can be a progressive process of wall thinning, positive remodeling and reduced LVEF in some subjects with FRDA. Indeed, there is evidence from a previous study that age is inversely associated with LV mass index in FRDA [6], suggesting either that higher LV mass index leads to early death, that increasing duration of the disease process leads to decreases in LV mass, or a combination of these mechanisms. There is also evidence from a small longitudinal study that progressive LV thinning occurs [12]. Whatever the process is, it was evident in the adult subjects with preservation of LVEF in the present study, where age was found to be an independent inverse correlate of LV mass index. Age was also an independent inverse correlate of LVEDL and LVEDV in the adults with FRDA, which is consistent with previous reports during aging in the healthy population [31, 45]. On the other hand, LVEDID does not change with age and LV end-diastolic external diameter increases with age during healthy aging [31], whereas age was inversely correlated with LVEDID and LV end-diastolic external diameter in adults with FRDA. Only a small group of subjects in the present study had a reduced LVEF at the time of their initial echocardiogram at our institution and further exploration of the nature, timing and causes of a reduced LVEF in FRDA will require larger numbers and longer-term follow up.

Comparison of LV echocardiographic findings in adult subjects with FRDA with age and sex-matched control subjects has been reported previously in subgroups of the current cohort who were part of studies which investigated LV and right ventricular long-axis function [7, 11]. Findings from these studies in the subjects with FRDA included increases in LV wall thickness, LV mass index and RWT, and a decrease in LVEDID compared to control subjects. In the present study, which examined and compared adults and children with FRDA, there was no control group included, and decisions about the presence of increases in RWT and LV mass index were determined by using published normal ranges, with adjustment for age group and sex as appropriate. The present study has substantially added to our previous findings in FRDA by demonstrating increases in age-normalized RWT, comparing different methods for LV mass indexation, showing that there are differences between children and adults and demonstrating that male sex is associated with increased LV size and mass index in both children and adults. In the present study we have also reported for the first time that there is a positive correlation between GAA1 and GAA2, and demonstrated that age and GAA1 (but not GAA2) have associations with LV variables in adults after adjustment for BSA and sex.

There are a number of limitations of this cross-sectional study. Early mortality will alter the makeup of the population of subjects with FRDA available to be included in a cohort study and this could distort our understanding of the LV structural changes in FRDA if a particular pattern of LV myocardial involvement was more likely to lead to premature death. Similarly the analysis of a group with normal LVEF has necessarily excluded a proportion of subjects who had previously progressed over time to have a reduced LVEF, and this could also confound analyses which include age or symptom duration. Unfortunately this limitation can only be fully addressed by long-term serial studies, preferably in which echocardiographic data was available prior to symptom onset. We have used an arbitrary definition for children as being up to the age of 18 years, but the definition of child versus adult has varied in population studies of LV structure. A diagnosis of reduced LVEF was based on a cutoff of <50% in the present study, but this number is also arbitrary, may not be ideal choice in FRDA, and based on data in population studies, possibly should vary depending on subject age and sex [46]. However, there is currently no evidence to support an alternative cutoff point for a normal LVEF in FRDA. The lack of positive findings with respect to associations of GAA1 in children compared to adults could be a false negative due to the smaller size of the group of children. However, there were no trends from the analysis in children to suggest that greater numbers were likely to have made a difference to the findings. Furthermore, as discussed above, there was evidence that some variables may be different in adults and children independently of other determinants. Information about smoking, alcohol intake, drug use, exercise and menopausal status was not collected and any LV effects of these factors could therefore not be evaluated in this study.

In conclusion, RWT and age-normalized RWT are the most sensitive echocardiographic measures for identifying LV involvement in FRDA and they have the advantage of being independent of sex and body size in both children and adults. Sex and body size are important determinants of most other measures of LV structure in FRDA, as they are in a healthy population, and it is therefore an important limitation of some previous FRDA studies that sex and/or body size were not taken into consideration in either defining abnormality or in assessing the relationship between GAA1 and LV structure. GAA1 is an independent determinant of both increased LV wall thickness and reduced LV size in adults, but not in children, and the existence of a difference between children and adults could be a contributing factor to previous discrepant findings with respect to the relationship of GAA1 with LV structure in FRDA. GAA2 is not an independent correlate of LV structural variables in either children or adults and previous positive findings with respect to GAA2 may just reflect the presence of a currently unexplained association between GAA2 and GAA1. Age is an independent predictor of smaller LV size and mass in adults, possibly reflecting in part the nature of cardiac disease progression in FRDA, however, additional longitudinal data will be required to further clarify this relationship, and also the determinants and time course of a reduced LVEF.

## Supporting information

S1 File Left ventricular structure in children and adults with FRDA. (XLS)

## Abbreviations

AOS: age at the onset of neurological symptoms
BSA: body surface area
FRDA: Friedreich ataxia
FXN: gene encoding for frataxin
GAA1: GAA repeat length within the shorter allele of the *FXN* gene
GAA2: GAA repeat length within the larger allele of the *FXN* gene
LV: left ventricular
LVEDID: left ventricular end-diastolic internal dimension
LVEDL: left ventricular end-diastolic length
LVEDV: left ventricular end-diastolic volume
LVEF: left ventricular ejection fraction
RWT: relative wall thickness
SWT: septal wall thickness

## References

[1] Delatycki MB, Corben LA (2012) Clinical features of Friedreich ataxia. J Child Neurol 27: 1133–1137. 10.1177/0883073812448230 22752493 PMC3674491

[2] Hewer RL (1968) Study of fatal cases of Friedreich's ataxia. BMJ 3: 649–652. 10.1136/bmj.3.5619.649 5673214 PMC1986520

[3] Tsou AY, Paulsen EK, Lagedrost SJ, Perlman SL, Mathews KD, Wilmot GR et al (2011) Mortality in Friedreich ataxia. J Neurol Sci 307: 46–49. 10.1016/j.jns.2011.05.023 21652007

[4] Isnard R, Kalotka H, Durr A, Cossee M, Schmitt M, Pousset F et al (1997) Correlation between left ventricular hypertrophy and GAA trinucleotide repeat length in Friedreichs ataxia. Circulation 95: 2247–2249. 10.1161/01.cir.95.9.2247 9142000

[5] Dutka DP, Donnelly JE, Nihoyannopoulos P, Oakley CM, Nunez DJ (1999) Marked variation in the cardiomyopathy associated with Friedreich's ataxia. Heart 81: 141–147. 10.1136/hrt.81.2.141 9922348 PMC1728941

[6] Meyer C, Schmid G, Gorlitz S, Ernst M, Wilkens C, Wilhelms I et al (2007) Cardiomyopathy in Friedreich's ataxia-assessment by cardiac MRI. Mov Disord 22: 1615–1622. 10.1002/mds.21590 17546670

[7] Mottram PM, Delatycki MB, Donelan L, Gelman JS, Corben L, Peverill RE (2011) Early changes in left ventricular long-axis function in Friedreich ataxia: relation with the FXN gene mutation and cardiac structural change. J Am Soc Echocardiogr 24: 782–789. 10.1016/j.echo.2011.04.004 21570254

[8] Weidemann F, Rummey C, Bijnens B, Stork S, Jasaityte R, Dhooge J et al (2012) The heart in Friedreich ataxia: Definition of cardiomyopathy, disease severity, and correlation with neurological symptoms. Circulation 125: 1626–1634. 10.1161/CIRCULATIONAHA.111.059477 22379112

[9] Regner SR, Lagedrost SJ, Plappert T, Paulsen EK, Friedman LS, Snyder ML et al (2012) Analysis of echocardiograms in a large heterogeneous cohort of patients with Friedreich ataxia. Am J Cardiol 109: 401–405. 10.1016/j.amjcard.2011.09.025 22078220

[10] St John Sutton M, Ky B, Regner SR, Schadt K, Plappert T, He J et al (2014) Longitudinal strain in Friedreich Ataxia: a potential marker for early left ventricular dysfunction. Echocardiography 31: 50–57. 10.1111/echo.12287 23834395

[11] Peverill RE, Donelan L, Corben LA, Delatycki MB (2018) Differences in the determinants of right ventricular and regional left ventricular long-axis dysfunction in Friedreich ataxia. PLoS ONE 13: e0209410 10.1371/journal.pone.0209410 PONE-D-18-20195 [pii]. 30596685 PMC6312254

[12] Weidemann F, Liu D, Hu K, Florescu C, Niemann M, Herrmann S et al (2015) The cardiomyopathy in Friedreich's ataxia—New biomarker for staging cardiac involvement. Int J Cardiol 194: 50–57. S0167-5273(15)01131-6 [pii]; 10.1016/j.ijcard.2015.05.074 26005806

[13] Campuzano V, Montermini L, Lutz Y, Cova L, Hindelang C, Jiralerspong S et al (1997) Frataxin is reduced in Friedreich ataxia patients and is associated with mitochondrial membranes. Hum Mol Genet 6: 1771–1780. 10.1093/hmg/6.11.1771 9302253

[14] Deutsch EC, Santani AB, Perlman SL, Farmer JM, Stolle CA, Marusich MF et al (2010) A rapid, noninvasive immunoassay for frataxin: utility in assessment of Friedreich ataxia. Mol Genet Metab 101: 238–245. 10.1016/j.ymgme.2010.07.001 20675166 PMC2996613

[15] Durr A, Cossee M, Agid Y, Campuzano V, Mignard C, Penet C et al (1996) Clinical and genetic abnormalities in patients with Friedreichs ataxia. N Engl J Med 335: 1169–1175. 10.1056/NEJM199610173351601 8815938

[16] Bit-Avragim N, Perrot A, Schols L, Hardt C, Kreuz FR, Zuhlke C et al (2001) The GAA repeat expansion in intron 1 of the frataxin gene is related to the severity of cardiac manifestation in patients with Friedreich's ataxia. J Mol Med 78: 626–632. 10.1007/s001090000162 11269509

[17] Pousset F, Legrand L, Monin ML, Ewenczyk C, Charles P, Komajda M et al (2015) A 22-Year Follow-up Study of Long-term Cardiac Outcome and Predictors of Survival in Friedreich Ataxia. JAMA Neurol 1–8 2444310 [pii]; 10.1001/jamaneurol.2015.1855 26414159

[18] Raman SV, Phatak K, Hoyle JC, Pennell ML, McCarthy B, Tran T et al (2011) Impaired myocardial perfusion reserve and fibrosis in Friedreich ataxia: a mitochondrial cardiomyopathy with metabolic syndrome. Eur Heart J 32: 561–567. 10.1093/eurheartj/ehq443 21156720 PMC3106287

[19] Rajagopalan B, Francis JM, Cooke F, Korlipara LV, Blamire AM, Schapira AH et al (2010) Analysis of the factors influencing the cardiac phenotype in Friedreich's ataxia. Mov Disord 25: 846–852. 10.1002/mds.22864 20461801

[20] Ribai P, Pousset F, Tanguy ML, Rivaud-Pechoux S, Le B, I, Gasparini F et al (2007) Neurological, cardiological, and oculomotor progression in 104 patients with Friedreich ataxia during long-term follow-up. Arch Neurol 64: 558–564. 10.1001/archneur.64.4.558 17420319

[21] Filla A, De Michele G, Cavalcanti F, Pianese L, Monticelli A, Campanella G et al (1996) The relationship between trinucleotide (GAA) repeat length and clinical features in Friedreich ataxia. Am J Hum Genet 59: 554–560. 8751856 PMC1914893

[22] Lamont PJ, Davis MB, Wood NW (1997) Identification and sizing of the GAA trinucleotide repeat expansion of Friedreich's ataxia in 56 patients. Clinical and genetic correlates. Brain 120 (Pt 4): 673–680. 9153129 10.1093/brain/120.4.673

[23] Montermini L, Richter A, Morgan K, Justice CM, Julien D, Castellotti B et al (1997) Phenotypic variability in Friedreich ataxia: role of the associated GAA triplet repeat expansion. Ann Neurol 41: 675–682. 10.1002/ana.410410518 9153531

[24] Monros E, Molto MD, Martinez F, Canizares J, Blanca J, Vilchez JJ et al (1997) Phenotype correlation and intergenerational dynamics of the Friedreich ataxia GAA trinucleotide repeat. Am J Hum Genet 61: 101–110. 10.1086/513887 9245990 PMC1715858

[25] Colan SD (2013) The why and how of Z scores. J Am Soc Echocardiogr 26: 38–40. 10.1016/j.echo.2012.11.005 23261367

[26] de Simone G, Daniels SR, Kimball TR, Roman MJ, Romano C, Chinali M et al (2005) Evaluation of concentric left ventricular geometry in humans: evidence for age-related systematic underestimation. Hypertension 45: 64–68. 10.1161/01.HYP.0000150108.37527.57 15557389

[27] de Simone G, Devereux RB, Daniels SR, Meyer RA (1995) Gender differences in left ventricular growth. Hypertension 26: 979–983. 10.1161/01.hyp.26.6.979 7490158

[28] Carroll JD, Carroll EP, Feldman T, Ward DM, Lang RM, McGaughey D et al (1992) Sex-associated differences in left ventricular function in aortic stenosis of the elderly. Circulation 86: 1099–1107. 10.1161/01.cir.86.4.1099 1394918

[29] Hees PS, Fleg JL, Lakatta EG, Shapiro EP (2002) Left ventricular remodeling with age in normal men versus women: novel insights using three-dimensional magnetic resonance imaging. Am J Cardiol 90: 1231–1236. 10.1016/s0002-9149(02)02840-0 12450604

[30] Lang RM, Badano LP, Mor-Avi V, Afilalo J, Armstrong A, Ernande L et al (2015) Recommendations for cardiac chamber quantification by echocardiography in adults: an update from the American Society of Echocardiography and the European Association of Cardiovascular Imaging. J Am Soc Echocardiogr 28: 1–39. S0894-7317(14)00745-7 [pii]; 10.1016/j.echo.2014.10.003 25559473

[31] Stoylen A, Molmen HE, Dalen H (2016) Importance of length and external diameter in left ventricular geometry. Normal values from the HUNT Study. Open Heart 3: e000465 10.1136/openhrt-2016-000465 openhrt-2016-000465 [pii]. 27752332 PMC5051511

[32] Devereux RB, Alonso DR, Lutas EM, Gottlieb GJ, Campo E, Sachs I et al (1986) Echocardiographic assessment of left ventricular hypertrophy: comparison to necropsy findings. Am J Cardiol 57: 450–458. 0002-9149(86)90771-X [pii]. 10.1016/0002-9149(86)90771-x 2936235

[33] de Simone G, Daniels SR, Devereux RB, Meyer RA, Roman MJ, de Divitiis O et al (1992) Left ventricular mass and body size in normotensive children and adults: assessment of allometric relations and impact of overweight. J Am Coll Cardiol 20: 1251–1260. 10.1016/0735-1097(92)90385-z 1401629

[34] Kampmann C, Wiethoff CM, Wenzel A, Stolz G, Betancor M, Wippermann CF et al (2000) Normal values of M mode echocardiographic measurements of more than 2000 healthy infants and children in central Europe. Heart 83: 667–672. 10.1136/heart.83.6.667 10814626 PMC1760862

[35] Peverill RE, Chou B, Donelan L, Mottram PM, Gelman JS (2016) Possible mechanisms underlying aging-related changes in early diastolic filling and long axis motion—Left ventricular length and blood pressure. PLoS ONE 11: e0158302 10.1371/journal.pone.0158302 PONE-D-16-02781 [pii]. 27351745 PMC4924872

[36] Evans-Galea MV, Carrodus N, Rowley SM, Corben LA, Tai G, Saffery R et al (2012) FXN methylation predicts expression and clinical outcome in Friedreich ataxia. Ann Neurol 71: 487–497. 10.1002/ana.22671 22522441

[37] Pfaffenberger S, Bartko P, Graf A, Pernicka E, Babayev J, Lolic E et al (2013) Size matters! Impact of age, sex, height, and weight on the normal heart size. Circ Cardiovasc Imaging 6: 1073–1079. CIRCIMAGING.113.000690 [pii]; 10.1161/CIRCIMAGING.113.000690 24014823

[38] Kou S, Caballero L, Dulgheru R, Voilliot D, De SC, Kacharava G et al (2014) Echocardiographic reference ranges for normal cardiac chamber size: results from the NORRE study. Eur Heart J Cardiovasc Imaging 16: 680–690. jet284 [pii]; 10.1093/ehjci/jet284 24451180 PMC4402333

[39] Pettersen MD, Du W, Skeens ME, Humes RA (2008) Regression equations for calculation of z scores of cardiac structures in a large cohort of healthy infants, children, and adolescents: an echocardiographic study. J Am Soc Echocardiogr 21: 922–934. 10.1016/j.echo.2008.02.006 18406572

[40] Cantinotti M, Scalese M, Murzi B, Assanta N, Spadoni I, De Lucia V et al (2014) Echocardiographic nomograms for chamber diameters and areas in Caucasian children. J Am Soc Echocardiogr 27: 1279–1292. S0894-7317(14)00586-0 [pii]; 10.1016/j.echo.2014.08.005 25240494

[41] Rinaldi C, Tucci T, Maione S, Giunta A, De Michele G, Filla A (2009) Low-dose idebenone treatment in Friedreich's ataxia with and without cardiac hypertrophy. J Neurol 256: 1434–1437. 10.1007/s00415-009-5130-6 19363628

[42] Plehn JF, Hasbani K, Ernst I, Horton KD, Drinkard BE, Di Prospero NA (2018) The Subclinical Cardiomyopathy of Friedreich's Ataxia in a Pediatric Population. J Card Fail 24: 672–679. S1071-9164(17)31221-6 [pii]; 10.1016/j.cardfail.2017.09.012 28986271

[43] Haland TF, Hasselberg NE, Almaas VM, Dejgaard LA, Saberniak J, Leren IS et al (2017) The systolic paradox in hypertrophic cardiomyopathy. Open Heart 4: e000571 10.1136/openhrt-2016-000571 openhrt-2016-000571 [pii]. 28674623 PMC5471858

[44] Sacca F, Puorro G, Antenora A, Marsili A, Denaro A, Piro R et al (2011) A combined nucleic acid and protein analysis in Friedreich ataxia: implications for diagnosis, pathogenesis and clinical trial design. PLoS ONE 6: e17627 10.1371/journal.pone.0017627 21412413 PMC3055871

[45] Peverill RE (2019) Aging and the relationships between long-axis systolic and early diastolic excursion, isovolumic relaxation time and left ventricular length-Implications for the interpretation of aging effects on e`. PLoS ONE 14: e0210277 10.1371/journal.pone.0210277 PONE-D-18-21676 [pii]. 30615676 PMC6322720

[46] Cheng S, Fernandes VR, Bluemke DA, McClelland RL, Kronmal RA, Lima JA (2009) Age-related left ventricular remodeling and associated risk for cardiovascular outcomes: the Multi-Ethnic Study of Atherosclerosis. Circ Cardiovasc Imaging 2: 191–198. 10.1161/CIRCIMAGING.108.819938 19808592 PMC2744970

